# Supplementary material for: Inhibitory peptidergic modulation of C. elegans serotonin neurons is gated by T-type calcium channels
Source: eLife. 2017 Feb 6;6:e22771. doi: 10.7554/eLife.22771 (PMC5330680; doi:10.7554/eLife.22771)
Supplement: Figure 7—source code 1. — DOI: http://dx.doi.org/10.7554/eLife.22771.022 [file elife-22771-fig7-code1.zip › Zang_et_al_Figure_7_Source_1_source_code_for_calcium_imaging_analysis/Zang_et_al_Figure_7_Source_1a_baseCorrectLocalMins.rtf]

function [ fCorr, correction] = baseCorrectLocalMins( t, fRaw, window )%baseCorrectLocalMins segments a time series (t,fRaw) into chunks of %'window' samples, finds local min in each chunk, fits a 1st order polynomial to%the local mins and subtracts this from fRaw     %t is "datapoint"; fRaw is "FQx_adj”, which is ROI-background (i.e.: raw data after background correction)%   t is a vertical vector of time stamps; fRaw is a vertical vector of background-adjusted fluorescence signals %   t and fRaw must be of the same length; 'window' is the size of the%   chunk in terms of how many data points, not time. For example, if%   'window' is 50, then the time series will be broken into chunks of 50%   data points and the number of local minima will be number of samples in%   fRaw/50.%   function returns a baseline-subtracted trace 'fCorr' and the baseline%   itself.%   to compute df/f: (fRaw-correction)./correction %   note: this is also fCorr./correction global numChunks; % how many chunks will fRaw be turned intoglobal numSamples; % size of fRawglobal tBase; % the times at the center of each chunkglobal fBase; % the minimum value in each chunk, which we will use as baseline  global p; % cfit object  figure; plot(t,fRaw,'-k'); % plot raw tracehold; numSamples = size(fRaw,1);numChunks = floor(numSamples/window); tBase = zeros(numChunks,1);fBase = zeros(numChunks,1); chunkFirst = 1;chunkLast = chunkFirst+window-1;counter = 1;  while (counter <= numChunks)    fBase(counter) = min(fRaw(chunkFirst:chunkLast));    tBase (counter) = t(round((chunkFirst+chunkLast)/2));    counter = counter+1;    chunkFirst = chunkLast+1;    chunkLast = chunkFirst+window-1;end plot(tBase, fBase, 'or'); % plot points used to infer baseline p = fit(tBase, fBase, 'poly1'); correction = p(t);plot(t,correction, '-r'); fCorr = fRaw-correction;     plot(t,fCorr, '-b'); % plot corrected trace%therefore, you will generate a plot with 2 lines. This will give you “gut check” to make sure no fundamental changes are occurring with the code. To get a variable with the data dF/F, type into command line >>fCorr./correction.This variable will give you the dF/F plot. You can then use this as in input into “findAbsValCumSum” to find the cumulative sum of your dF/F plot.You can also follow this script with “calciumOutput”, and it will compute dF/F and the cumulative sum of the signal for you.   end
