# Supplementary material for: Inhibitory peptidergic modulation of C. elegans serotonin neurons is gated by T-type calcium channels
Source: eLife. 2017 Feb 6;6:e22771. doi: 10.7554/eLife.22771 (PMC5330680; doi:10.7554/eLife.22771)
Supplement: Figure 7—source code 1. — DOI: http://dx.doi.org/10.7554/eLife.22771.022 [file elife-22771-fig7-code1.zip › Zang_et_al_Figure_7_Source_1_source_code_for_calcium_imaging_analysis/Zang_et_al_Figure_7_Source_1b_findAbsValCumSum.rtf]

function FQx_abs_cumsum=findAbsValCumSum(FQx_dF_over_F, FQx_cumsum)%Written with David Schoppik, Neuroscience Institute, NYU School of Medicine. This function is if I've done baseCorrectLocalMins and already found%dF_over_F and cumsum. It calculates the absolute value of the cumulative sum to give the total activity over time.diff(FQx_dF_over_F);plot(abs(diff(FQx_dF_over_F)))%abs is absolute value, so it will count peaks and valleys FQx_abs_cumsum=cumsum(abs(diff(FQx_dF_over_F)));    %calculate the cumulative sum of the areas of the peaks and valleys % option:sum(abs(diff(FQ1078_C_3_neurite_bundle_adj(1:100))))    %looking only at certain points in the data. Can use this to look only at peaks and exclude fluctuating baseline.figureplot(FQx_abs_cumsum)figureplot(FQx_dF_over_F)figureplot(FQx_cumsum)end
