# Supplementary material for: Inhibitory peptidergic modulation of C. elegans serotonin neurons is gated by T-type calcium channels
Source: eLife. 2017 Feb 6;6:e22771. doi: 10.7554/eLife.22771 (PMC5330680; doi:10.7554/eLife.22771)
Supplement: Figure 7—source code 1. — DOI: http://dx.doi.org/10.7554/eLife.22771.022 [file elife-22771-fig7-code1.zip › Zang_et_al_Figure_7_Source_1_source_code_for_calcium_imaging_analysis/Zang_et_al_Figure_7_Source_1c_calciumOutput.rtf]

function [FQx_dF_over_F, FQx_abs_cumsum ] = calciumOutput(FQx_fCorr,FQx_correction)%Written with David Schoppik, Neuroscience Institute, NYU School of Medicine.This function is for when I've already done baseCorrectLocalMins and need%to figure out the cumulative sum of the absolute value of the dF/FFQx_dF_over_F=FQx_fCorr./FQx_correction;plot(FQx_dF_over_F)diff(FQx_dF_over_F);figureplot(abs(diff(FQx_dF_over_F)))%abs is absolute value, so it will count peaks and valleys FQx_abs_cumsum=cumsum(abs(diff(FQx_dF_over_F)));    %cumulative sum of the areas of the peaks and valleys % option:sum(abs(diff(FQ1078_C_3_neurite_bundle_adj(1:100))))    %looking only at certain points in the data. Can use this to look only at peaks and exclude fluctuating baseline.figureplot(FQx_abs_cumsum) end
