# Supplementary material for: Inhibitory peptidergic modulation of C. elegans serotonin neurons is gated by T-type calcium channels
Source: eLife. 2017 Feb 6;6:e22771. doi: 10.7554/eLife.22771 (PMC5330680; doi:10.7554/eLife.22771)
Supplement: Supplementary file 1. — DOI: http://dx.doi.org/10.7554/eLife.22771.024 [file elife-22771-supp1.docx]

**Table T1**

| Strains | p-value |
| --- | --- |
| *egl-6(gf)* vs wild type | 2.2e-16 |
| *egl-6(gf)* vs *egl-6(gf) cca-1(n5209)* | 2.2e-16 |
| *egl-6(gf) cca-1(n5209)* vs wild type | 0.003 |
| *cca-1(n5209)* vs wild type | 0.6808 |
| *egl-47(gf)* vs *egl-47(gf); cca-1(n5209)* | 0.002 |
| *egl-10(lf)* vs *egl-10(lf); cca-1(n5209)* | 1.24e-6 |
| *egl-6(gf)* vs *egl-6(gf) cca-1(n5209)/cca-1*(+) | 1.43e-9 |
| *egl-6(gf) cca-1(n5209)* vs *egl-6(gf) cca-1(ad1650)* | 2.2e-16 |
| *cca-1(n5209)* vs *cca-1(ad1650)* | 5.36e-10 |
| *goa-1(lf); egl-6(gf)* vs *goa-1(lf); egl-6(gf) cca-1(n5209)* | 2.8e-10 |
| *flp-10(Δ) flp-17(Δ); egl-6(gf)* vs  *flp-10(Δ) flp-17(Δ); egl-6(gf) cca-1(n5209)* | 6.22e-15 |
| *egl-1(gf)* vs *egl-1(gf); cca-1(n5209)* | 0.891 |
| *egl-6(gf) cca-1(n5209); wzEx125[ cca-1::GFP]* vs  *lin-39(n709); egl-6(gf) cca-1(n5209); wzEx125[ cca-1::GFP]* | 2.4e-4 |
| *egl-6(gf) cca-1(n5209)* vs *egl-6(gf) cca-1(n5209)*  with *egl-6a::cca-1 RNAi* (HSN-specific knockdown) | 2.2e-16 |
| *egl-6(gf) cca-1(n5209)* vs *egl-6(gf) cca-1(n5209)*  with *ceh-24::cca-1 RNAi* (VM-specific knockdown) | 0.33 |
| *egl-6(gf) cca-1(n5209)* vs *egl-6(gf) cca-1(n5209)*  with *cca-1::cca-1 RNAi* (VC-specific knockdown) | 0.41 |
| *egl-6(gf) cca-1(n5209)* with HSN-specific knockdown  vs wild type with HSN-specific knockdown | 6.62e-7 |

p-values were computed using an Asymptotic Wilcoxin Mann-Whitney Rank Sum Test. Presented values are uncorrected for multiple comparisons.
