# Supplementary material for: Inhibitory peptidergic modulation of C. elegans serotonin neurons is gated by T-type calcium channels
Source: eLife. 2017 Feb 6;6:e22771. doi: 10.7554/eLife.22771 (PMC5330680; doi:10.7554/eLife.22771)

File: pKEZ18_KZ36.ab1 Run Ended: 2015/8/26 14:45:52 Signal G:176 A:158 C:185 T:150

Sample: pKEZ18_KZ36 Lane: 67 Base spacing: 14.490241 1197 bases in 13763 scans

N NNNN NNTNNNNNN NTGNN NGCG ATG AAGGGNCTCTCGTCCTCATTCTCTGTCTGGTAATAAGGCTCCAGGTCCACGCTCAGGGGGAGGCTGAAGTTCTCGGGGAGGAAGCACCGGTTGCGAAGC

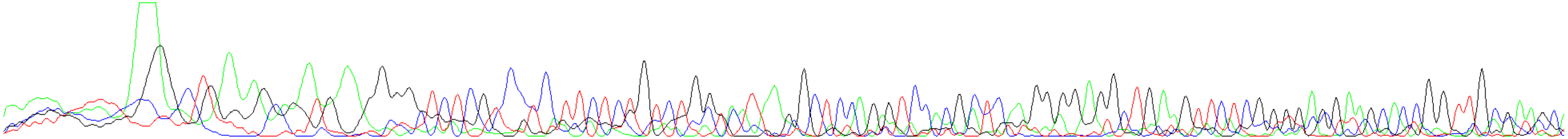

AGTCCTGCCCACAGCTGGACGCCCACGATGCCAAAGATGAAAAAGACGAAGAAACAGAGCAGCAGGACGTTGCCCAGCATAGACAAGGTGTCCAGCAGTAATGTGACGAGAATGCGCATGCTGGGCACCCGG

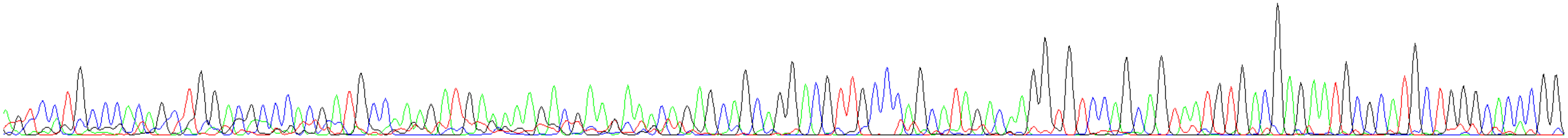

TTAATGGCCCTGAGCGGTCGCAGCACACGGACTGTCCTGACTGCGGAGAAGCTGACGTTCTGCAGGTCCAGCGAATACTCCAGCATCCCTGCAATGACAATGAAAAAGTCAAGCCGGTTCCAAGTGTCTCCCAGGT

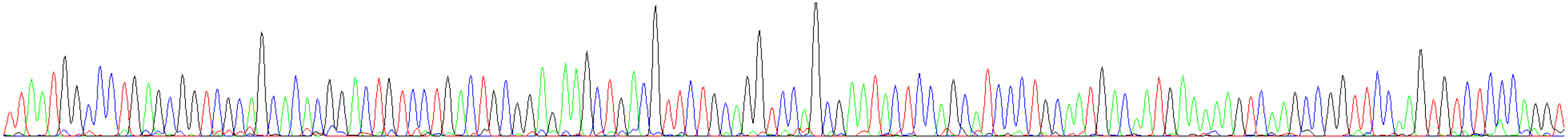

AACATTTCTTCCCAAAGATGCCCAAGGCCACCATCTTCACCACCATTTCCACAGCAAAGAAGGCAAAGATGAAGTCATCGAAGGCCTGCAGGATCCGGCAGCGCTGGGAGTCACAGGCAATGTCCTCACACGGCCTG

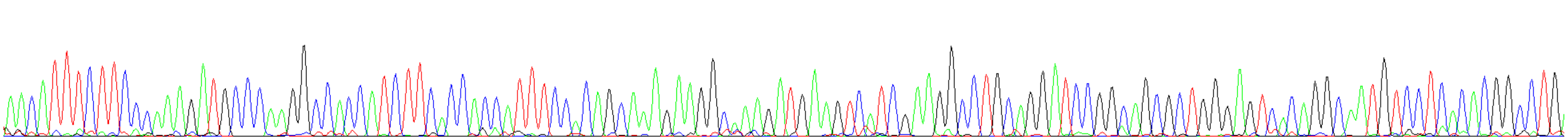

AACATACCCAGAGTCACACAGTTGAGAAGAATGACCAGCATACTGACTCGCTCGAACCACGGGTTACAGACCGTGCGGAGACACCAGCTCCGCGGGCGGCTGTCCTGGCTCAAGTAGAAGAAAACCACCGGGGCTAGCG

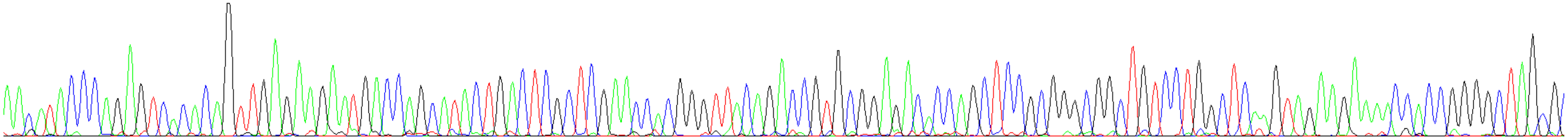

File: pKEZ18_KZ36.ab1　　*Run Ended: 2015/8/26 14:45:52*　　*Signal G:176 A:158 C:185 T:150*

Sample: pKEZ18_KZ36　　*Lane: 67*　　*Base spacing: 14.490241*　　*1197 bases in 13763 scans*　　

670 680 690 700 710 720 730 740 750 760 770 780 790 800
CCGGGACGGCAGCCCCTCCGCCTCGGATCCGCGCTGCCCGGGCCTTTTCCTCGACCCGGCCCTGCCGNCCCCGGCCCGGACGGTCGTTGAGCTGCGTGAGCTACGGGGCTGTCCCGACTCCTCGGCGCCGCT

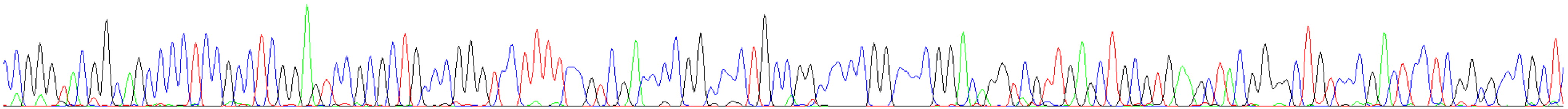

810 820 830 840 850 860 870 880 890 900 910 920
CCATCCTCTCCTCGCCATCTCTGGCAGCCGGGCCCGGGGGTCCGAAAGGGCGAGCGGAGCCCTCAAGGAGGGTCTCACGCACGGGGACCGGTGGGGAGATGTGGAACCGATTCTCGTGAGGGGATT

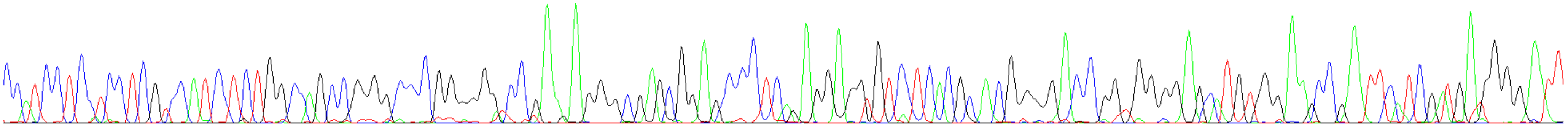

930 940 950 960 970 980 990 1000 1010 1020 1030 1040 1050
GACTGCAAAGTGACGTTATCTGACTTTGCAAAAANCAGCAGCTGGTACGACTCGAATATTCGCCTAAGGAGCGCATACATTCCTGCCGCGTTTACACGCCTGCTGGAAACCTGCGTACCACTTAT

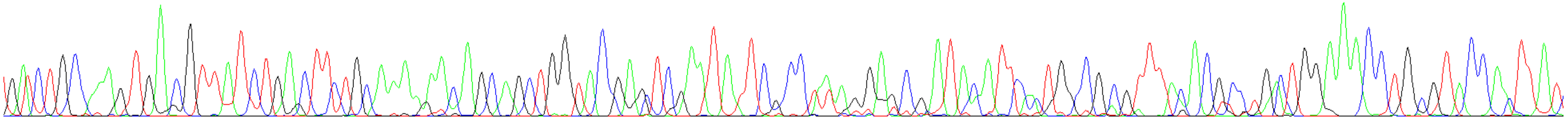

1060 1070 1080 1090 1100 1110 1120 1130 1140 1150 1160 1170
CCNTGCACANTCCCTTCGCAGTGCGAANCAAANGCCGCNNNCGCTTCCANNTNCNNNCTGATGCAAGGNCCGAGNGATTTCCTACATTGGCGATTCACCGATGGNCCTCANANTCGCTNNCNA

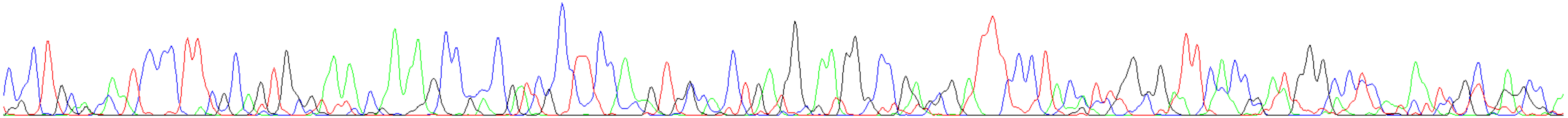

1180 1190
ANTACCACCNACCGCAACCGTA

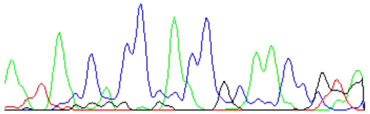

Supplement: Supplementary file 2. — DOI: http://dx.doi.org/10.7554/eLife.22771.025 [file elife-22771-supp2.zip › plasmid_sequences/pKEZ18_KZ36.pdf]
