# Supplementary material for: Inhibitory peptidergic modulation of C. elegans serotonin neurons is gated by T-type calcium channels
Source: eLife. 2017 Feb 6;6:e22771. doi: 10.7554/eLife.22771 (PMC5330680; doi:10.7554/eLife.22771)
Supplement: Supplementary file 3. — DOI: http://dx.doi.org/10.7554/eLife.22771.026 [file elife-22771-supp3.docx]

**Strains used in this study:**

**JD21** *cca-1(ad1650)*

**FQ226** *cca-1(n5209)*

**FQ46** *egl-6(n529) cca-1(n5209)*

**MT1222** *egl-6(n592)*

**MT1082** *egl-1(n487)*

**MT1443** *egl-10(n692)*

**MT2248** *egl-47(n1081)*

**MT16698** *goa-1(n1134); egl-6(n592)*

**FQ37** *flp-10(n4543) flp-17(n4894); egl-6(n592)*

**FQ232** *egl-6(n592) cca-1(ad1650)*

**FQ604** *wzEx125[Prom_cca-1(3kb)_::GFP]*

**FQ666** *egl-10(n692); cca-1(n5209)*

**FQ707** *egl-47(n1081); cca-1(n5209)*

**FQ757** *lin-39(n709); egl-6(n592) cca-1(n5209); wzEx125[Prom_cca-1(3kb)_::GFP]*

**FQ810** *egl-1(n487); cca-1(n5209)*

**FQ862** *egl-6(n592) cca-1(n5209); wzEx125[Prom_cca-1(3kb)_::GFP]*

**FQ863** *goa-1(n1134); egl-6(n592) cca-1(n5209)*

**FQ892** *flp-10(n4543) flp-17(n4894); egl-6(n592) cca-1(n5209)*

**FQ992** *wzEx227[Prom_egl-6a_::GCaMP6f Prom_unc-122_::mCherry]*

**FQ1078** *cca-1(n5209); wzEx227[Prom_egl-6a_::GCaMP6f Prom_unc-122_::mCherry]*

**FQ1079** *egl-6(n592); wzEx227[Prom_egl-6a_::GCaMP6f Prom_unc-122_::mCherry]*

**FQ1081** *egl-6(n592) cca-1(n5209); wzEx227[Prom_egl-6a_::GCaMP6f Prom_unc-122_:mCherry]*

**FQ1194** *lin-15AB(n765); wzEx335[Prom_ceh-24_::GCaMP6f; lin-15(+)]*

**FQ1201** *lin-15AB(n765) egl-6(n592); wzEx335[Prom_ceh-24_::GCaMP6f; lin-15(+)]*

**FQ1202** *lin-15AB(n765) cca-1(n5209); wzEx335[Prom_ceh-24_::GCaMP6f; lin-15(+)]*

**FQ1203** *lin-15AB(n765) egl-6(n592) cca-1(n5209); wzEx335[Prom_ceh-24_::GCaMP6f; lin-15(+)]*

**FQ1267** *wzEx343[Prom_cca-1_::GFP; Prom_flp-17_::mStrawberry]*

**FQ1348** *wzEx355[Prom_cca-1_::GFP; Prom_egl-6a_::mStrawberry]*

**FQ1357** *egl-6(n592) cca-1(n5209); wzEx360[Prom_ceh-24_::cca-1 RNAi*]

**FQ1364** *egl-6(n592) cca-1(n5209); wzEx367[Prom_cca-1(3kb)_::cca-1 RNAi*]

**FQ1447** *lin-39(n709); wzEx355[Prom_cca-1_::GFP; Prom_egl-6a_::mStrawberry]*

**FQ1468** *egl-6(n592) cca-1(n5209); wzEx402[Prom_egl-6a_::cca-1 RNAi; Prom_flp-17_::mStrawberry ]*

**FQ1649** *wzEx402[Prom_egl-6a_::cca-1 RNAi; Prom_flp-17_::mStrawberry]*

**FQ1650** *wzEx402[Prom_egl-6a_::cca-1 RNAi; Prom_flp-17_::mStrawberry]*
