# Supplementary material for: Inhibitory peptidergic modulation of C. elegans serotonin neurons is gated by T-type calcium channels
Source: eLife. 2017 Feb 6;6:e22771. doi: 10.7554/eLife.22771 (PMC5330680; doi:10.7554/eLife.22771)
Supplement: Supplementary file 4. — DOI: http://dx.doi.org/10.7554/eLife.22771.027 [file elife-22771-supp4.docx]

**Primers and plasmids used in this study:**

| Genotyping Primer Name | Primer Sequence 5’🡪 3’ |
| --- | --- |
| *egl-6(wt)* forward | TTCACAACGACGATCGTTGGCG |
| *egl-6(n592)* forward | CATTCACAACGACGATCGTTGCCA |
| *egl-6* universal reverse | ATTAACGACGGAAGTAGTTTGAACAC |
| *cca-1(wt)* forward | AAACATAAAAGAAGCACATTTCCAAGCATGTG |
| *cca-1(n5209)* forward | AAAAAACATAAAAGAAGCACATTTCCAAGCATGTA |
| *cca-1* universal reverse | ACTAACAACTCATGTTCGGCCGTTGATAAA |
| *lin-39(wt)* forward | GGATGACGGTGATGTTGATGTGGTCTTC |
| *lin-39 (n709)* forward | GATGACGGTGATGTTGATGTGGTCAGT |
| *lin-39* universal reverse | CATCTCTATCGTCCTCTTATTCTATTCCCTCATCC |
| *goa-1(wt)* forward | CCGTTCATCAACTCTAGCGCCACG |
| *goa-1(n1134)* forward | CACCGTTCATCAACTCTAGCGCCAAA |
| *goa-1* universal reverse | TTAATAATTCAAATCGATCAAACGAAA |

| Constructed Plasmid Name | Description |
| --- | --- |
| pSAZ66 | *Prom_cca-1(3kb)::_GFP* |
| pNR559 | *Prom_egl-6a_::GCaMP6f* |
| pKEZ52 | *Prom_ceh-24_::GCaMP6f* |
| pEH30 | *Prom_egl-6a_::mStrawberry* |
| pEH4 | *cca-1 fosmid::GFP* |
| pKEZ93 | *Prom_egl-6a_::cca-1* RNAi antisense |
| pKEZ67 | *Prom_egl-6a_::cca-1* RNAi sense |
| pKEZ66 | *Prom_ceh-24_::cca-1* RNAi antisense |
| pKEZ65 | *Prom_ceh-24_::cca-1* RNAi sense |
| pKEZ63 | *Prom_cca-1(3kb)_::cca-1* RNAi antisense |
| pKEZ64 | *Prom_cca-1(3kb)_::cca-1* RNAi sense |
| pKEZ18 | Cav3.1(P262S) in pGEMHEA |

| Injected Constructs | Injection Concentration |
| --- | --- |
| *Prom_ttx-3_::mStrawberry* | 40 ng ul^-1^ |
| *lin-15*(+) | 15-30 ng ul^-1^ |
| *Prom_unc-122_::mCherry* | 20 ng ul^-1^ |
| *Prom_egl-6a_::GCaMP6f* | 100 ng ul^-1^ |
| *Prom_ceh-24_::GCaMP6f* | 100 ng ul^-1^ |
| *Prom_flp-17_::mStrawberry* | 30 ng ul^-1^ |
| *Prom_cca-1_::GFP* fosmid amplicon | 40 ng ul^-1^ |
| RNAi constructs | 100 ng ul^-1^ |
