# Supplementary material for: Inhibitory peptidergic modulation of C. elegans serotonin neurons is gated by T-type calcium channels
Source: eLife. 2017 Feb 6;6:e22771. doi: 10.7554/eLife.22771 (PMC5330680; doi:10.7554/eLife.22771)
Supplement: Supplementary file 5. — DOI: http://dx.doi.org/10.7554/eLife.22771.028 [file elife-22771-supp5.docx]

**RNAi lines were generated using the following seeds:**

***cca-1* sense: 5’-** cgcaattgaacatgttaaagagggtgaacttgatgaagaagaagagacagaagaaggtccca ccactcaaataccagacgggcatggtggtattaaacggttatccatgcaggttctggaacaagaattaatcgaagtcgagagacatttggaagaaagatatcggagggcaagcgagtgtctcggcggagaacttcagcctttgaatcccggagagatcgaagatctagacgatcccgagttcagaccacggagtagatcacatagaccacgagcaagaacaaacagtgcgttgagcaataaaagccgtggatcacacaagtctgcttta **– 3’**

***cca-1* antisense: 5’-** taaagcagacttgtgtgatccacggcttttattgctcaacgcactgtttgttcttgctcgtggt ctatgtgatctactccgtggtctgaactcgggatcgtctagatcttcgatctctccgggattcaaaggctgaagttctccgccgagacactcgcttgccctccgatatctttcttccaaatgtctctcgacttcgattaattcttgttccagaacctgcatggataaccgtttaataccaccatgcccgtctggtatttgagtggtgggaccttcttctgtctcttcttcttcatcaagttcaccctctttaacatgttcaattgcg **– 3’**
